# Trailblazers in foregut surgery: Peter Nau, MD (University of Iowa) interviews David Rattner, MD (Harvard University)

**DOI:** 10.1007/s00464-025-11828-9

**Published:** 2025-06-06

**Authors:** Peter Nau, David Rattner

**Affiliations:** 1 University of Iowa, Iowa City, USA; 2 Massachusetts General Hospital, Boston, MA USA

After nearly 30 years of laparoscopic anti-reflux surgery, there are so many fellows and residents that have been trained to perform these procedures. Over the course of these years, fellows and residents continue to provide ongoing training to others in anti-reflux procedures. What is clear from SAGES Masters sessions in Foregut Surgery at the annual meetings, is there are certain technical aspects of the procedure that are passed along through the ongoing Minimally Invasive Surgical training. SAGES has identified several Trailblazers in Foregut Surgery, and the following are transcripts of conversations with the founding experts in anti-reflux surgery. These were video discussions that occurred in 2019, and SAGES would like to memorialize this series as they offer truly valuable experiences.

All readers will find these interviews interesting, from experts to trainees. We may be able to identify through these transcripts whose style of foregut surgery we follow. The Trailblazers were interviewed by members of the SAGES Foregut Committee during the 2019 SAGES Annual Meeting.

**Peter Nau: My name is Peter Nau. I am an Associate Professor of Surgery at the University of Iowa Hospitals and Clinics. I’m here with Dr. David Rattner, who is a Professor of Surgery at Harvard Medical School and Chief of the Division of Gastrointestinal and General Surgery at Massachusetts General Hospital. Our interview is a part of SAGES’ Trailblazers in Foregut Surgery series. Dr. Rattner, thank you for taking the time to meet with us today. I have several questions for you.**

**You’ve been a big mentor for me and someone whom I’ve modeled my life after, not only in the clinic but also outside of the hospital. One of the things that I wonder when I think about my mentors is: whom did you look up to when you were growing up? Was it an esophageal surgeon? Or was it your dad? Who led you to become who you are today?**

David Rattner: My dad was a physician and that probably helped me to understand what it takes to have a career in medicine. But I wouldn’t say that he was my mentor.

My surgical mentor definitely was Andy Warshaw, who is a true general surgeon but became very famous for his work in pancreatic surgery. I think that we both had a bit of a rebel streak in us. When I was a resident working with him, we loved to find some dogmatic statement or principle and see whether we could knock it down. That rebel streak of his resonated with me. And he was a tremendous surgeon. He is a tremendous friend, and I would say that he was my mentor at Massachusetts General.

**Did you stumble onto esophageal surgery? You do a little bit of esophageal surgery and a little bit of colorectal surgery, which is very uncommon. Is that something that you always envisioned yourself doing? Or did that evolve as you went along and your practice matured? How did you see yourself at the start of your career compared to where you are now?**

When I was a resident, I was sent to the laboratory to do gastric physiology. I spent 2 years in the laboratory of William Silen, who was a famous GI physiologist and Chief of Surgery at Beth Israel. As I was finishing my residency, Dr. Gerry Austen recruited me to stay in Boston. I never imagined I would stay there.

And so, I started on that pathway. But when I was doing my super chief year, I was interested in pancreatic disease and pancreatic problems. So, there was some dissonance between what I was being recruited for and what I was interested in. And during the first couple of years, my own research in gastric physiology didn’t progress as rapidly as I had wished.

I was talking to Andy quite a bit, and I started to do some projects with him. My lab focus shifted from gastric physiology to acute pancreatitis. That’s how our relationship began. We’ve had a fair amount of success in laboratory and clinical publications.

But the key step occurred when I was presenting a poster on acute pancreatitis at, I believe, the American Pancreatic Association, next to another young surgeon named Karl Zucker, whom I’d befriended. Karl and I had a social relationship as well. We were out for dinner, and he said, “You should come down to Baltimore next week. I'm going to do something crazy, and I think you'd enjoy it.” It turned out that he was doing laparoscopic cholecystectomy. So, I went and watched him do a couple of operations.

I came back to Boston and said, “Andy, I'm going to do this. We have to do this.” Andy said, “That’s crazy.” And I said, “No. If you trust me, I think this is going to be really big.” At the end of the day, he backed me up, and we got started on doing laparoscopic cholecystectomy. We were among the first surgeons—if not the first—in Boston to do that procedure.

Through Karl Zucker, I immediately jumped into laparoscopic Nissen fundoplications. At about that point, one of my co-resident fellows, Charlie Ferguson, returned. Charlie and I began doing some laparoscopic Nissen fundoplications together, and off we went.

I guess it’s a matter of being in the right place at the right time. For example, John Hunter had been my surgical endoscopy fellow when I was the super chief. I had a good relationship with John, who told me about SAGES around the time when these things were happening. So, I developed a squad (in modern parlance) of good friends, including Nathaniel Soper, John Hunter, Lee Swanstrom, Joe Peatland, and Bruce Schirmer—a bunch of us who were the early folks doing laparoscopic surgery.

And it goes back to Andy Warshaw. People said, “You shouldn’t do this,” and we got a lot of grief about it—people saying that it wasn’t safe, it wasn’t this, and it wasn’t that. But the patients were flocking to us. We were a big threat to them because we were trying to take away their practice. But the fact of the matter is that we were doing 10 or 11 laparoscopic cholecystectomies per day. People were just beating down the doors to have this operation. In all fairness, I think that we did it safely. It’s not the way we would do it nowadays, but—at least among my inner circle of colleagues there—we were thoughtful as we went about this. That’s how it all started.

In terms of how the practice ended up being so much foregut and esophageal, I think it’s a matter of what you get known for. When the laparoscopic Heller myotomy came along, I was the only guy in the hospital who was doing it.

The other factor—which is very different today than it was when I finished my super chief in 1985—is that at that time, we did a lot of thoracic surgery. I served as the acting thoracic chief resident for several months. So, I had plenty of thoracic experience. I wasn’t afraid to operate on the chest. It was just a different kind of training. It seemed very natural to me. As long as my results were good, nobody bothered me about doing a thoracotomy. So that’s how it went.

**This is interesting. When you read the articles about the people who started laparoscopy, it’s just crazy to think about the process that they went through to teach themselves how to do it. And you’re one of those early folks. Was there a time when you thought, “This isn’t what I thought it would be. It’s not going to take off?” Or did you always see laparoscopy heading to where it is today as the gold standard?**

I didn’t see everything going as far as it has gone, to be honest with you. There’s a huge difference in terms of outcomes with laparoscopic versus open cholecystectomy and that differential was fortunate. The evolution was very patient driven. In the beginning, laparoscopic cholecystectomies were much more challenging, and I think other people deserve a lot of credit for making that happen.

I think we’ve almost gone to the opposite extreme today. The residents whom we see now have a lot of trouble with open surgery. I never thought I’d say it, but it is important to have that tool—the technical aspects of open surgery—in your toolkit. When I see people spend 6 or 7 h doing an operation robotically or laparoscopically, it drives me and people my age a bit crazy, because we could make an incision and usually complete things more efficiently.

In fact, one of the last papers I’ve written had to do with open surgery for complicated hiatal hernia repairs. The results in those patients are spectacularly good for very difficult operations. So, I think that, if anything, the evolution of laparoscopy has gone further than I would have imagined. It’s unclear what the place of open surgery will be in future. But, at least in my hands, there’s still a place for it.

**A laparoscopic cholecystectomy is always going to be a bit more straightforward. But when you talk about the beginning of laparoscopy in the complex foregut, were you just taking the open operation and replicating it laparoscopically? What was that learning curve like, since it’s a distinctly different operation in laparoscopy? Did you have to invent on the fly? What was that process like?**

It was a little bit of trial and error. Don’t forget that at that time, there was no good digital imaging. We were passing VCR cassettes back and forth to each other—or eventually DVDs, when DVDs came along. We would go to meetings and share our experiences. There was a lot of sharing: “I did this, and I did that” or “You did this, but why don’t you try it this way?” or “I’m going to fly out to Portland and come to see what you do. You should come to Boston and see what I do.” The internet existed, but no one used the web as we do now. It was a “clubby” environment, as I would describe it. If you were in the club, people would tell you their secrets and then you could try them out.

In the field of foregut surgery, the development of the harmonic scalpel was a tremendously important enabling step. When we began, we’d have to place these little clips on the short gastrics, and sometimes they’d fall off. It wasn’t easy to stop the bleeding. Or you could have a splenectomy. As the equipment improved and the energy devices, in particular, improved, it became a much easier operation. In fact, it’s much easier to do a laparoscopic Nissen than an open Nissen because of issues related to exposure.

**What do you think has been the most important development? When I was in residency, one of my staff used to talk about Bovie being the groundbreaking thing for surgery because of energy devices and how it changed the way we achieve hemostasis. From a laparoscopic standpoint, is the key development the optics? Or harmonic energy devices? Can you identify 1 thing that most facilitated the growth, or are there many things?**

I think the key development is the energy devices, whether harmonic or bipolar or whatever. Those devices make a tremendous difference and allow you to do things that sometimes, even in open surgery, would be very difficult to do with a clamp and a LigaSure.

But the stapling technology was also critical in making a leap forward. All the metabolic and bariatric surgeries are enabled by the advances in stapling devices.

**Something that I’ve always been interested in—and that drew me to your fellowship, in particular—is the idea of innovation. Dr. Melvin was someone who taught me as a resident. He was one of the big doctors in robotics. And you were involved very early on in research.**

**What do you think is the next big thing? Is it already out there? Is it POEM? Is it LINX? Are these devices or approaches going to be anything? I think that at some point, innovation becomes difficult because of societal, financial, or medical-legal factors in the environment.**

I think that if you’re looking at what happened in the past, you’re looking in the wrong place. That’s not innovation. That’s why disruptive innovation is unknowable. You can do all the market research that you want, but even market research looks backward and not necessarily forward.

I think that the next big innovation is going to be based on big data, machine learning, artificial intelligence, genetics, targeted therapies, or targeted surgery. I think that’s where the opportunity is. I don’t think that anyone has quite put it all together yet. But it seems that those are the technologies that will change the way that today’s 25- and 30-year-old surgeons and surgical trainees will practice. I would bet that what you will be doing when you finish your career 20 or 30 years from now doesn’t yet exist. What I did later in my career didn’t exist when I trained, and the pace of change is ever increasing.

**I think about my practice… It’s so little, with a robot. A lot of the people who trained me have always done it with the straight sticks. I don’t know whether I’m fighting something or whether the pendulum has swung so far with the robot that it’s going to swing back—or whether there will be some iteration of the robot that’s different from what we have now.**

Right now, the robot is a master–slave system. It has good optics, but it doesn’t do anything that you couldn’t do yourself with your hands. We haven’t taken advantage of the computer and the robot, at least not as far as I’m concerned. Once we do capitalize on the computing ability and the ability to learn or interpret data that you can’t process fast enough with your brain, then I think we’re going to have something special.

So, there’s no question in my mind that computer-assisted surgery, in some form, is important and will be a key part of the future. Whether it’s the da Vinci system—I don’t think so, to be honest with you. But the da Vinci system married to good use of computational power—then I think we’re going to have something.

**I think that there are 2 unique aspects about you. One of those parts is the clinical aspect. The other thing that I've always admired—you know, we went to see Bruce Springsteen when I was a fellow, and I got to see the Bruins games with you—is that you’ve always had a healthy grasp on when to work and when to play. Who taught you that? Was that something that resulted from your wife saying, “Listen, make time for the family?” Or was that something that you saw with your parents? Where did you learn how to play hard and work hard? I think that’s one of the most important things that I've learned from you.**

That’s not from my wife. She works harder than I do, and God bless her. It’s just my nature. I have trouble doing things halfway. In college, if we partied, we partied hard; if we worked, we worked hard. I guess I am still immature enough that when I party, I party hard. That’s just the way I roll.

**Thanks for taking the time to meet with me. You’ve been a huge part of so many people’s lives, whether it’s your fellows or your surgical trainees. As you think back and look ahead over the next few years, it will be interesting to see all these people as they mature. I think you will see more and more people who are becoming chiefs and medical directors. You’ve influenced a lot of people, and it’s been great being trained by you.**

Thank you, Peter, for your kind words. I immensely enjoyed our year together.

